# Stat3 Inhibitors TTI-101 and SH5-07 Suppress Bladder Cancer Cell Survival in 3D Tumor Models

**DOI:** 10.3390/cells13171463

**Published:** 2024-08-31

**Authors:** Surya P. Singh, Gopal Pathuri, Adam S. Asch, Chinthalapally V. Rao, Venkateshwar Madka

**Affiliations:** 1 Center for Cancer Prevention and Drug Development, Stephenson Cancer Center, Hem-Onc Section, Department of Medicine, University of Oklahoma Health Sciences Center, Oklahoma City, OK 73104, USA; surya-singh@ouhsc.edu (S.P.S.); gopal-pathuri@ouhsc.edu (G.P.); cv-rao@ouhsc.edu (C.V.R.); 2 Stephenson Cancer Center, Hem-Onc Section, Department of Medicine, University of Oklahoma Health Sciences Center, Oklahoma City, OK 73104, USA; adam-asch@ouhsc.edu

**Keywords:** bladder cancer, STAT3 inhibitor, apoptosis, preclinical, 3D models

## Abstract

Bladder cancer (BCa) is one of the most lethal genitourinary malignancies owing to its propensity for recurrence and poor survival. The biochemical pathway, signal transducer and activator of transcription 3 (STAT3), has gained significance as a molecular pathway that promotes proliferation, invasion, and chemoresistance. In this study, we explored the targeting of STAT3 with TTI-101 and SH5-07 in BCa and elucidated the mechanisms in three-dimensional (3D) spheroid and organoid models. We optimized the growth of spheroids from human, rat, and mouse BCa cell lines (J82, NBT-II, and MB49 respectively) and organoids from BBN (N-butyl-N-(4-hydroxybutyl)-nitrosamine)-induced rat bladder tumors. Cell viability was assessed using MTT and trypan blue assays. Intracellular ATP production, ROS production, and calcium AM (CA)/EtBr staining were used to measure the spheroid and organoid inhibition and mitochondrial function. Western blot analysis was performed to evaluate the pharmacodynamic markers involved in cell proliferation, apoptosis, cancer stem cells (CSCs), and STAT3 signaling in BCa. We found that targeting STAT3 (using TTI-101 and SH5-07) significantly reduced the proliferation of BCa spheroids and organoids, which was accompanied by decreased expression of pSTAT3, Cyclin D1, and PCNA. Our data also demonstrated that treatment with STAT3 inhibitors induced ROS production and cell death in BCa spheroids and organoids. STAT3 inhibition-induced cell death was associated with the activation of caspase 3/7 and PARP cleavage. Moreover, TTI-101 and SH5-07 target cancer stem cells by downregulating the expression of CD44 and CD133 in 3D models. This study provides the first evidence for the prevention of BCa with small-molecule inhibitors TTI-101 and SH5-07 via suppression of CSCs and STAT3 signaling.

## 1. Introduction

Bladder cancer (BCa) is the 10th most common malignancy worldwide [1]. In the United States, 82,290 new cases and 16,710 cancer-related deaths are projected to have occurred in 2023 [2]. Despite recent advances in its treatment, including chemotherapy, immunotherapy, and surgery, the survival rate of patients with metastatic BCa remains poor. Over the past few years, new strategies have emerged for the management of advanced BCa. For instance, the FDA has approved the immune checkpoint inhibitors PD1/PD-L1 for the treatment of BCa. Unfortunately, many patients do not respond to these immune checkpoint inhibitors [3]. Therefore, platinum-based chemotherapy remains the most effective treatment for patients with advanced metastatic BCa [4]. However, after treatment, many patients experience a significant rate of tumor recurrence. Therefore, there is an urgent need to improve existing BCa management options and develop novel treatment strategies to intercept or delay BCa progression and improve survival rates.

STAT3 (signal transducer and activator of transcription 3) proteins are intracellular transcription factors that regulate several cellular processes including proliferation, apoptosis, epithelial–mesenchymal transition, stemness, and immune escape. STAT3 activity is tightly regulated during normal physiology in nonmalignant cells, but it is chronically active in almost 70% of solid tumors [5,6]. STAT3 activation involves phosphorylation of Tyr705, a critical tyrosine residue that causes STAT3 to undergo homo- or heterodimerization, which facilitates its nuclear localization and DNA binding [7]. Consequently, downstream gene transcription is initiated, which controls essential biological functions.

Studies have reported that bladder malignancies are associated with constitutive STAT3 activation [8]. A growing body of evidence indicates that persistence of cancer stem cells (CSCs) is responsible for tumor development, recurrence, metastasis, chemoresistance, and radio-resistance [9]. Activated STAT3 requires co-expression of Oct3/4 and Nanog, markers of pluripotent stem cells [10]. As a result of these signaling pathways, CSCs markers such as CD44 are upregulated, which increases the niche of CSCs [11,12]. Furthermore, CD133 levels were positively correlated with poor prognosis and tumor growth in patients with BCa [13]. Therefore, STAT3 is considered a promising target for intercepting BCa recurrence and progression. A wide variety of STAT3 activation inhibitors have been reported, including peptides, small molecules, and oligonucleotides [14]. Among these, small molecule inhibitors such as GLG-302 [15], TTI-101 [16], and SH5-07 [17] are designed to abrogate the phosphorylation and dimerization of STAT3. SH5-07 is shown to inhibit the proliferation of breast cancer, human glioma, and prostate cancer cells by decreasing the expression of Bcl-2, Bcl-xL, Mcl-1, cyclin D1, and c-Myc [17]. Similarly, STAT3 inhibitor, TTI-101, has been shown to have potent anticancer effects in preclinical animal models [16]. Unfortunately, due to various limitations, only a few of these agents could progress to an early-phase clinical study.

Many preclinical drug development studies used cancer cell lines grown in 2D culture. Studies have shown that cancer cells exhibit altered biological characteristics, including genetic expression, biological activity, and loss of heterogeneity depending on culture conditions [18]. Thus, 2D in vitro models are unable to accurately depict the histopathological characteristics of human diseases, limiting the translational relevance of research findings using this system [19]. Owing to these differences, in addition to other limitations, only a few of these could progress to an early phase clinical study. Recently, three-dimensional in vitro models have gained popularity for drug development because they closely mimic in vivo environments under heterogeneous and physiological conditions to a certain extent [20]. To the best of our knowledge, this is the first study to examine the anticancer mechanisms of STAT3 inhibition by TTI-101 and SH5-07 on BCa spheroids and organoids.

## 2. Materials and Methods

### 2.1. Cell Culture

Human bladder carcinoma (J82; ATCC Cat # HTB-1), rat bladder carcinoma (NBT-II; ATCC Cat # CRL-1655), and mouse bladder carcinoma (MB49; Accegen Cat # ABC-TC2235S) cell lines were purchased from commercial sources. J82 and NBT-II cells were grown in Eagle’s minimum essential medium, and MB49 cells were grown in Dulbecco’s modified Eagle medium supplemented with 10% fetal bovine serum and 1% penicillin–streptomycin at 37 °C in a 95% humidified atmosphere with 5% CO_2_ and maintained by subculturing the cells twice a week. STAT3 inhibitors (GLG 302, TTI-101, and SH5-07) were obtained from the NCI-DCP repository.

### 2.2. MTT Assay

J82, NBT-II, and MB49 cells were seeded at a density of 8 × 10^3^ cells per well in a 96-well culture plate (Corning Costar, Corning, NY, USA) using standard protocol [21]. After 24 h, cells were treated with STAT3 inhibitors (GLG-302, TTI-101, and SH5-07) at concentrations ranging from 0 to 50 µM for 24 and 48 h, followed by MTT (Sigma-Aldrich, St. Louis, MO, USA) assays. A standard microplate reader was used to detect the optical density (OD) at 570 nm (Scientific Multiskan MK3, Thermo Fisher Scientific, Waltham, MA, USA).

### 2.3. Cell Growth and Death Assay

Briefly, 1 × 10^5^ cells were seeded in each 60 mm culture dish and incubated overnight. Cells were treated with GLG-302, TTI-101, and SH5-07 at concentrations of 0, 6, 12.5, 25, and 50 µM for 48 h. After the treatment, cells were collected by trypsinization and washed twice with phosphate-buffered saline (PBS). Then, cells were gently mixed with trypan blue dye (Sigma-Aldrich, USA) and counted using a TC20 automated cell counter (Bio-Rad, Hercules, CA, USA).

### 2.4. Spheroids Culture

J82, NBT-II, and MB49 spheroids were generated using a simple and repeatable procedure that yielded a single spheroid in a single well. BCa cells were grown as a monolayer, trypsinized, and washed. Cells were concentrated and seeded at 1.5 × 10^3^ cells/well in 96-well ultra-low attachment (ULA) plates with 200 µL of complete medium, allowing the cells to assemble into compact 3D aggregates. Every second day, the medium was carefully replaced with fresh medium without disturbing the spheroid growth. A bright-field microscope was used to monitor spheroid growth over a period of 12 days.

### 2.5. Organoids Culture

The carcinogen-induced BCa rat model development was approved by the IACUC of the University of Oklahoma HSC (Protocol # 20-079). BCa was induced in female F344 rats by oral administration of bladder-specific carcinogen N-Butyl-N-(4-hydroxybutyl) nitrosamine (BBN) following standard protocol [22]. Bladder tumor tissue from the BBN-induced rat BCa model was collected at termination and cut into small pieces (1–2 mm) in PBS with a sterile surgical blade. Tumor pieces were placed in 1 mg/mL collagenase solution in advanced Dulbecco’s modified DMEM/F-12 media with ROCK inhibitor (MedChemExpress # Y-27632). Tissue was incubated at 37 °C for 30 min periods while shaking. Following incubation, the cell suspension was filtered through a 70-micron filter, and the cells were collected by centrifugation at 1800 rpm for 5 min. Then, 1 × 10^5^ cells were mixed with 1 mL of basement membrane extract (BME) and plated in individual wells of a pre-warmed 24-well plate. When the BME solidified, DMEM/F-12 medium supplemented with 100 ng/mL of FGF10 (Peprotech # 100-26), 25 ng/mL of FGF7 (Peprotech # 100-19), A83-01(MedChemExpress # HY-10432) and 500 nM of ROCK inhibitor (MedChemExpress # Y-27632,) and B27 (Gibco # 17504044) was added, and the cells were incubated in CO_2_ incubators. Bright-field microscopy was used to observe the growth of organoids after 24 h of incubation.

### 2.6. Growth and Viability Assay 

To measure the growth kinetics, spheroids were grown for 12 days on ULA plates without treatment. Images of spheroids and organoids were obtained using a microscope. ImageJ software ver. 1.53e (National Institutes of Health, Bethesda, MD, USA) was used to measure spheroid size. For viability assays, spheroids and organoids were grown in triplicate. Twenty-four hours after seeding, spheroids and organoids were treated with dimethyl sulphoxide (DMSO) or STAT3 inhibitor for 144 h. Intracellular adenosine triphosphate (ATP) concentration was used as a measure for cell viability and measured using CellTiter-Glo^®^ 3D (Promega, Madison, WI, USA). For ATP measurement, 100 mL of CellTiter-Glo^®^ 3D reagent was added to each well of culture media. The contents were mixed for five minutes to induce cell lysis and incubated at room temperature for 25 min, and then luminescence was recorded. Dose–response curves were plotted using GraphPad Prism 8 (GraphPad Software, San Diego, CA, USA).

### 2.7. Calcein AM/EtBr Dual Staining 

Spheroids and organoids were generated as described above; 24 h after cell seeding, drug treatment was performed for 6 days. At the end of the treatment period, the spheroids were rinsed with 1× PBS, pH 7.4, and then incubated with calcein-AM (Invitrogen™ # C1430) and Ethidium bromide (EtBr; Invitrogen™ # 15585011) solutions for 30 min at final concentrations of 2 mM and 4 mM, respectively. After incubation, the spheroids were washed with 1× PBS twice and images were captured with a fluorescence microscope. 

### 2.8. MitoSOX Staining

A MitoSOX staining assay was performed to measure the production of mitochondrial reactive oxygen species (ROS) in BCa spheroids and organoids. Spheroids and organoids were incubated with STAT 3 inhibitors for six days. After incubation, spheroids and organoids were stained with 5 μM MitoSOX (Invitrogen™ # M36008) red dye, protected from light for 30 min. Spheroids and organoids were washed with PBS to remove any residual dye and images were recorded using fluorescence microscopy.

### 2.9. MitoTracker and Caspase 3/7 Staining

BCa spheroids and organoids were treated with a known concentration of STAT3 inhibitors (TTI-101 and SH5-07) for six days. Later, they were incubated with a staining buffer containing MitoTracker (Invitrogen™ # M7512) red and CellEvent Caspase-3/7 (Invitrogen™ # C10423) green. Fluorescence images captured using a fluorescence microscope were processed using ImageJ software.

### 2.10. Cell Lysis and Immunoblotting

At the completion of the treatment, BCa cells, spheroids, and organoids were lysed with radioimmunoprecipitation assay (RIPA) cell lysis buffer (Thermo Fisher Scientific, USA) containing protease and phosphatase inhibitors. Protein was quantified using the Pierce™ BCA protein assay kit (Thermo Fisher Scientific, USA); 10–20 µg of protein per lane was resolved on an 8–10% SDS-PAGE gel and then transferred onto a PVDF membrane. Membranes were incubated with primary and secondary antibodies to determine the expression of various proteins. The following primary antibodies were used: STAT3 (CST-30835, 1:1000), p-STAT3 (CST-9145, 1:1000), Cyclin D1(CST-55506, 1:1000), proliferating cell nuclear antigen (PCNA; CST-13110, 1:1000), caspase 3 (CST-9662, 1:1000), cleaved PARP (CST-5625, 1:1000), CD44 (CST-37259, 1:1000), and CD133 (AB-A0219, 1:1000) and Rabbit secondary antibody (CST-7074, 1:10000). Enhanced chemiluminescence was detected using a Gbox instrument (Syngene, Bangalore, India). ImageJ software was used to scan the bands, and densitometry was performed.

### 2.11. Statistical Analysis

All experiments were performed thrice with duplicate samples from each treatment group. Data are presented as mean + SEM. Significant differences between treatments are analyzed using students’ *t*-test with Welch’s correction. The level of significance is presented by * *p* < 0.05, ** *p* < 0.001, *** *p* < 0.0001.

## 3. Results

### 3.1. p-STAT3 Inhibition Suppresses the Proliferation of BCa Cells

**Figure 1:**
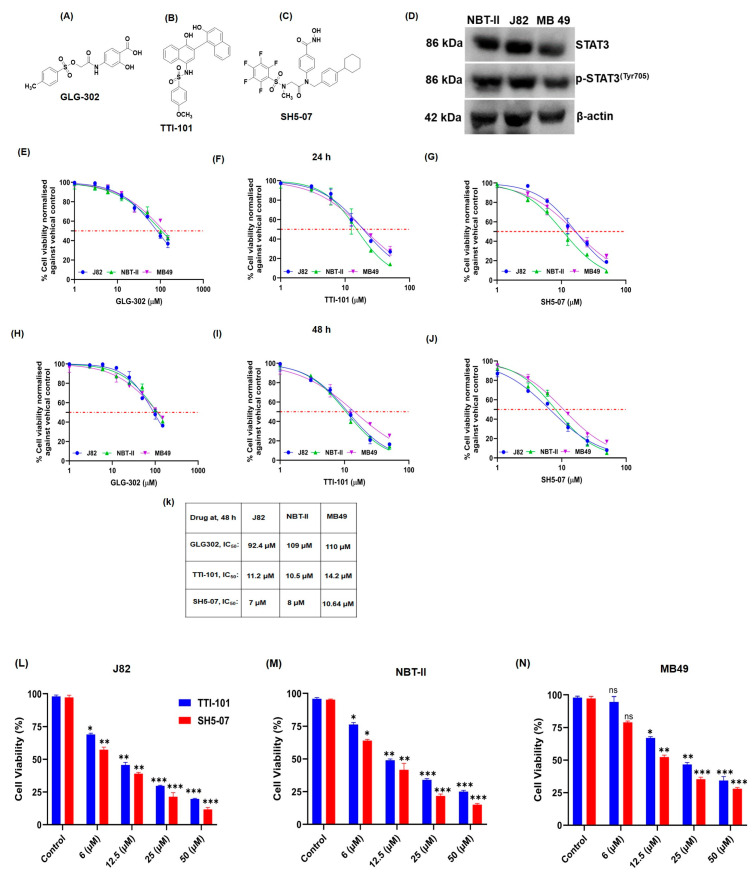
Effect of STAT3 inhibitors on the growth and viability of BCa J82, NBT-II, and MB49 cells. Chemical structure of STAT3 inhibitors (**A**) GLG-302, (**B**) TTI-101, and (**C**) SH5-07. (**D**) Basal expression levels of STAT3 and pSTAT3 in BCa cell line (NBT-II, J82, and MB49). (**E**–**J**) The MTT assay was used to determine the viability of BCa cells at 24 h and 48 h, as shown in graphs. (**K**) IC50 values of compounds on NBT-II, J82, and MB49 cells at 48 h of treatment. Cells were cultured with various doses of STAT3 inhibitors (1–50 µM) and processed for the MTT experiment, as detailed in the Materials and Methods Section. BCa cells were treated with different concentrations of STAT3 inhibitors (0, 6, 12.5, 25, and 50 µM) for 48 h and live cells were counted using the trypan blue dye exclusion method (**L**–**N**). The results are representative of three independent experiments. Error Bars = Standard error of mean (SEM); * *p* < 0.05; ** *p* < 0.001; *** *p* < 0.0001.

STAT3 inhibitors GLG-302, TTI-101, and SH5-07, (Figure 1A–C) were used in this study. Protein expression analysis indicated that all the BCa cell lines [human (J82), rat (NBT-II), and mouse (MB49) origin] used here have basal expression of STAT3 and pSTAT3^Y705^, enabling us to evaluate STAT3 inhibitor activity (Figure 1D). To study the effect of STAT3 inhibition on BCa cell growth and survival, selected STAT3 inhibitors were evaluated in vitro by treating the BCa cell lines with these drugs at 0, 1, 3, 6, 12.5, 25, and 50 µM concentration for 24 and 48 h. The results indicated that TTI-101 and SH5-07 (IC50 ranges 7–14.2 µM) had a strong inhibitory effect on the proliferation of J82, NBT-II, and MB49 cells as compared to GLG-302, which had a moderate effect even at higher concentrations (Figure 1E–J). Further, cell viability analysis using a Trypan-blue assay of drug-treated BCa cell lines also confirmed a strong anticancer effect of STAT3 inhibitors (Figure 1L–N). Based on these findings, TTI-101 and SH5-07 were selected for further investigation on BCa using spheroid and organoid models.

### 3.2. p-STAT3 Inhibitors Suppressed BCa Growth in 3D Spheroid Models

**Figure 2:**
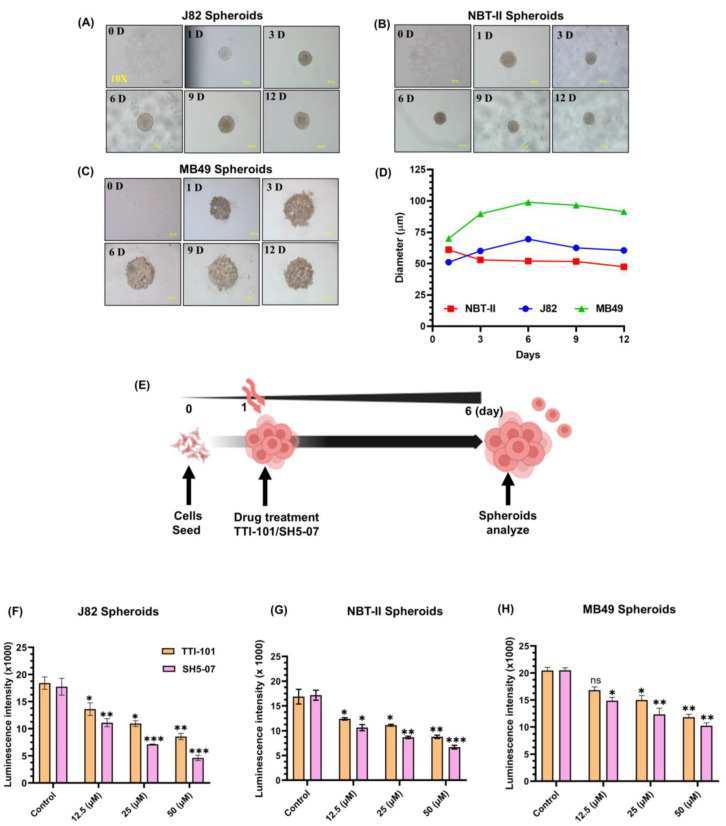

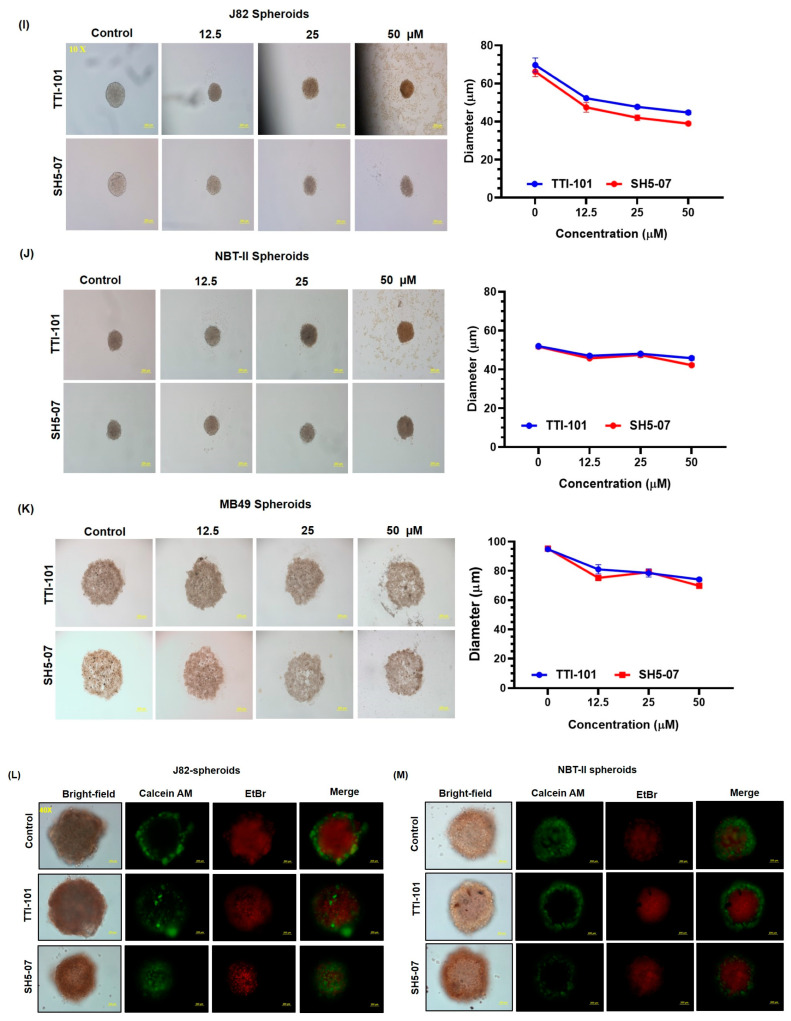
Effect of STAT3 inhibitors on proliferation of BCa spheroids. Representative images of spheroids generated using J82 (**A**), NBT-II (**B**), and MB49 (**C**) BCa cell lines. All images were captured at a 10X magnification. Growth curves of spheroid sizes (**D**). Schematic diagram illustrating treatment of spheroids with STAT3 inhibitors (**E**). Intracellular ATP content in (**I**–**K**) spheroids measured by luminescence on day six (**F**–**H**). Treatment with STAT3 inhibitors decreased the BCa spheroids size; bar represents 200 µM diameter (**I**–**K**). Spheroids were stained with calcein AM for live cells (green) and EtBr for dead cells (red). Representative images of control vs. treated BCa spheroids (**L**,**M**). All experiments were performed in triplicate (*n* = 3). Values are expressed as the mean ± SEM. Significance is indicated by * *p* < 0.05, ** *p* < 0.001, and *** *p* < 0.0001.

Prior to drug testing, we optimized spheroid growth using J82, NBT-II, and MB49 BCa cell lines. For this, cells were seeded into ULA plates, and the spheroid formation and size were monitored at regular intervals for 12 days under a microscope. All three cell lines formed condensed and spherical spheroids (Figure 2A–C). During 14 days of observation, J82 and NBT-II spheroids increased up to 6 days, and it later remained stable; however, MB49 cells formed less compact spheroids (Figure 2D). The spheroids generated were treated with STAT3 inhibitors (TTI-101 and SH5-07) and their viability was determined by measuring their diameters and intracellular ATP content (Figure 2E). We observed a significant decrease in the spheroid viability (20–40%, *p* < 0.0001) (Figure 2F–H) as well as a decrease in BCa spheroid size in response to TTI-101 and SH5-07 treatment (Figure 2I–K). MB49 spheroids showed decreased proliferation (Figure 2H) (10–25%, *p* < 0.001); however, the spheroid diameter was less effected due to diffused growth characteristics (Figure 2K). Thus, MB49 spheroids were excluded from further experiments. Calcein-AM and EtBr staining indicated that TTI-101 and SH5-07 treatment enhanced cell death in BCa spheroids compared to the control (Figure 2L–M). These results demonstrate that TTI-101 and SH5-07 suppressed the growth of BCa spheroids.

### 3.3. p-STAT3 Inhibition Enhanced Apoptosis via Mitochondrial ROS Induction and Inhibited Stemness in BCa Spheroids

**Figure 3:**
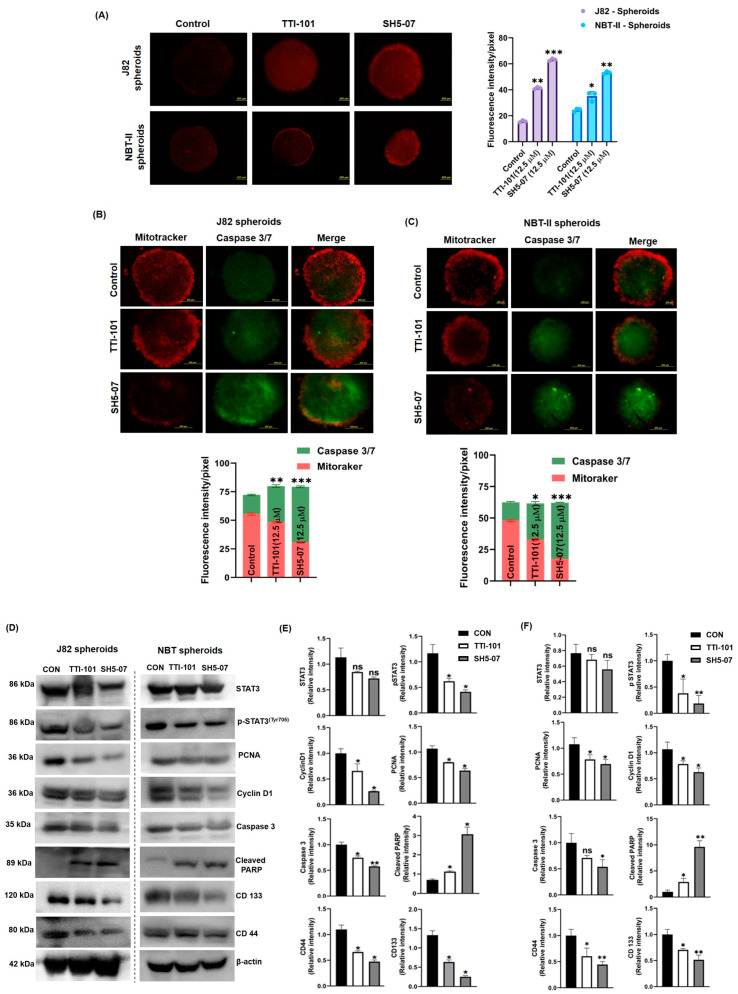
Effect of STAT3 inhibitors on apoptosis induction in BCa spheroids. BCa spheroids were treated with STAT3 inhibitors, stained with MitoSOX, and visualized under a fluorescent microscope (**A**). Spheroids stained with MitoTracker (red) and CellEvent Caspase-3/7 (green). Images were acquired using 50X objectives (**B**,**C**). Protein lysate was prepared from control (CON) and STAT3 inhibitor-treated BCa spheroids (TTI-101- or SH5-07(12.5 µM)) for 6 days to determine the expression of STAT3, pSTAT3, Cyclin D1, PCNA, Caspase 3, cleaved PARP, CD133, and CD44. β-actin was used as a loading control (**D**). The intensity of the indicated protein expression in J82 spheroids (**E**) and NBT-II spheroids (**F**) was quantified using ImageJ software. Data represent the average of three independent experiments and are reported as the mean ± SEM (* *p* < 0.05, ** *p* < 0.001, *** *p* < 0.0001).

Activation of ROS is known to interfere with apoptosis induction; therefore, we examined whether mitochondrial ROS played any role in TTI-101- and SH5-07-induced apoptosis in BCa spheroids. The ROS generation within BCa spheroids was quantified by staining BCa spheroids with MitoSOX Red dye. It was observed that SH5-07 induced significant mitochondrial ROS production in both BCa spheroids as compared to TTI-101 (Figure 3A). Further, MitoTracker and caspase-3/7 staining was used to determine apoptosis induction in BCa spheroids in response to TTI-101 and SH5-07 treatment. In live cells, the mitochondrial staining dye MitoTracker diffuses passively across the plasma membrane and accumulates in active mitochondria, whereas caspase-3/7 stains apoptotic cells. The results showed that treatment with TTI-101 and SH5-07 significantly decreased MitoTracker fluorescence intensity while increasing caspase-3/7 intensity in both BCa spheroids (Figure 3B,C), suggesting that STAT3 inhibitors induced apoptosis in BCa spheroids. Further, we found that STAT3 inhibitors (TTI-101 and SH5-07) significantly reduced the expression of pSTAT3^Y705^ and cell proliferation-related proteins PCNA and cyclin D1. In addition, J82 spheroids were more sensitive than NBT-II spheroids to both inhibitors (TTI-101 and SH5-07). Drug treatment also enhanced the expression of cleaved PARP apoptosis marker in both BCa spheroids. The stem cell marker proteins CD44 and CD133 were also downregulated by TTI-101 and SH5-07 treatments (Figure 3D). Collectively, these findings demonstrate that the STAT3 inhibitor SH5-07 more significantly decreased the growth of BCa via ROS-induced apoptosis and suppression of stemness compared to TTI-101.

### 3.4. p-STAT3 Inhibitors Suppressed Rat BCa Organoid Growth and Survival

**Figure 4:**
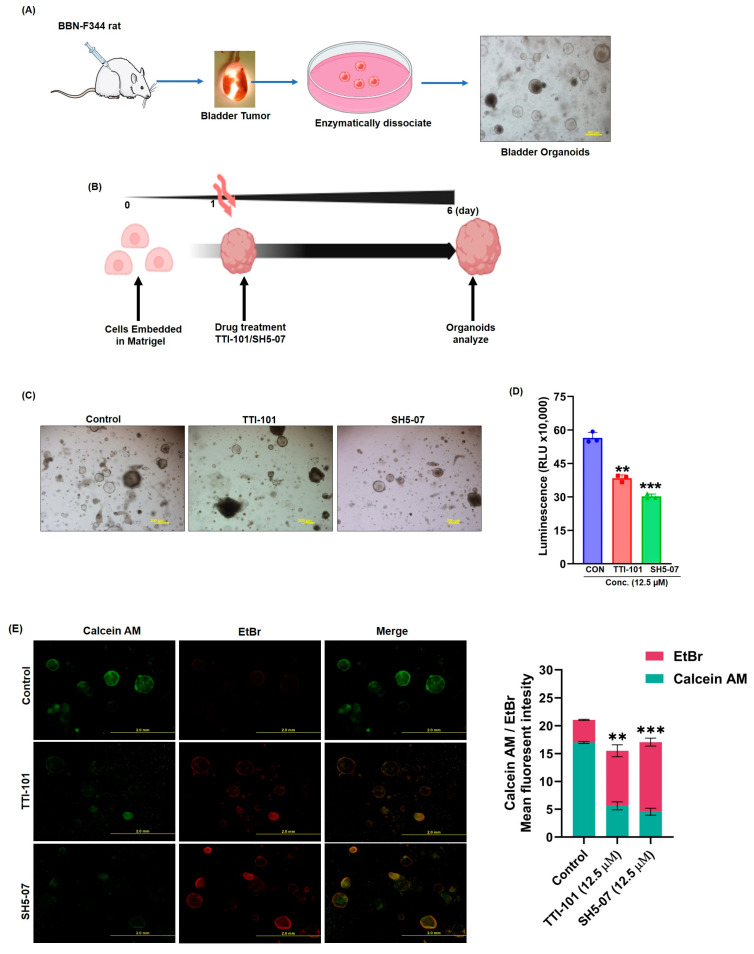
Effect of STAT3 inhibitors on BBN-induced rat tumor-derived BCa organoids. Schematic of organoid culture procedure from rat bladder tumors (**A**). Overview of the timeframe experiment (**B**). STAT3 inhibitors (TTI-101 and SH5-07) decreased organoid growth (**C**). Tumor cell proliferation in control (CON) and STAT3 inhibitor-treated organoids was measured using the CellTiter-Glo assay after six days of drug treatment (**D**). Assessment of STAT3 inhibitors on bladder organoids using ethidium homodimer (dead) and calcein AM (live) staining (**E**). Values are (*n* = 3) mean ± SEM (** *p* < 0.001, *** *p* < 0.0001).

Rat BCa organoids were established from freshly collected tumor tissues, as described in the Methods Section (Figure 4A). Next, we assessed the growth of organoids by measuring their viability in response to TTI-101 and SH5-07. Our data indicated that TTI-101 treatment reduced the viability organoids by 28% (*p* < 0.05) and the SH5-07 treatment reduced them by 31% (*p* < 0.05) (Figure 4B–D). Further, we determined the mode of cell death and morphological changes caused by TTI-101 and SH5-07 by staining the organoids with dead–live calcein-AM/EtBr. The results showed that TTI-101 and SH5-07 induced cell death in the treated organoids compared to the control (Figure 4E).

**Figure 5:**
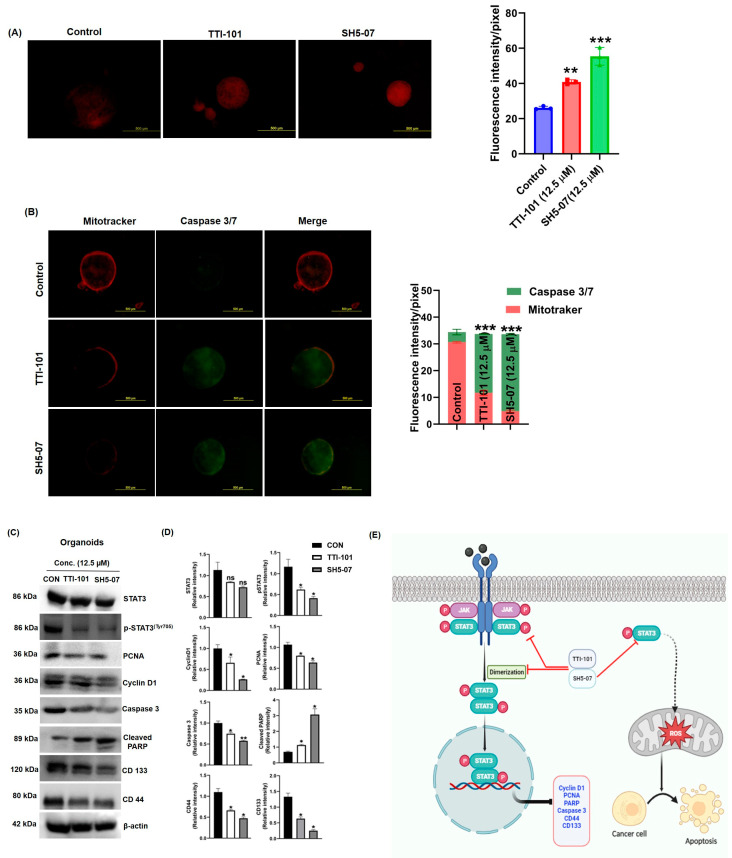
Effect of STAT 3 inhibitors on apoptosis induction in BCa organoids. Fluorescence staining of mitochondrial superoxide production after treatment with TTI-101 and SH5-07 at concentration of 12.5 µM for six days (**A**). After TTI-101 and SH5-07 treatment, organoids were stained with MitoTracker (red) and CellEvent Caspase-3/7 (green) to detect cell death within organoids (**B**). Protein lysate was prepared from control (CON) and STAT3 inhibitor-treated BBN-rat BCa organoids (TTI-101 or SH5-07 (12.5 µM)). STAT3, pSTAT3, Cyclind1, PCNA, caspase 3, cleaved PARP, and CD133 and CD44 protein expression levels were detected by immunoblotting. β-actin was used as a loading control (**C**). Densitometric analysis was performed to quantify the protein expression in the BCa organoids (**D**). Values are (*n* = 3) mean ± SEM (* *p* < 0.05, ** *p* < 0.001, *** *p* < 0.0001). Graphical summary of the molecular effects of the STAT3 inhibitors on BCa cells (**E**).

Both agents induced ROS generation; however, SH5-07 appeared to be more efficient than TTI-101 (Figure 5A). We also found that TTI-101 and SH5-07 treatment increased caspase3/7 activity and decreased MitoTracker intensity compared to untreated organoids (Figure 5B).

Additionally, we verified the effect of TTI-101 and SH5-07 treatment on the expression of proteins involved in proliferation, apoptosis, stem cells, and STAT3 signaling in BCa organoids. The results of Western blotting showed a decrease in the expression of pSTAT3^Y705^, the proliferation marker PCNA, and Cyclin D1, which correlated to the reduced BCa organoid size (Figure 5C). The apoptosis cleaved PARP marker proteins were significantly increased in BCa organoids following treatment with TTI-101 and SH5-07, while the stem cell markers CD44 and CD133 were significantly decreased (Figure 5C). These results imply that inhibition of STAT3 signaling, induction of apoptosis, and decreased stem cell markers could be one of the anticancer mechanisms of TTI-101 and SH5-07 (Figure 5E).

## 4. Discussion

Recently, significant attention has been paid to developing more predictable cellular models for in vitro screening of anticancer agents. Three-dimensional culture models mimic a microenvironment like that found within in vivo tumors by maintaining the phenotypes and functions of native cells. Mostly, 3D BCa spheroids and organoid models are typically produced from commercial cell lines and bladder tumor tissues using an ultra-low-attachment plate and Matrigel, respectively [23,24]. Here, we established a spheroid and organoid culture method for BCa cells and a BBN-rat BCa model to investigate the molecular mechanisms of small-molecule p-STAT3 inhibitors. The culture approach is based on earlier established spheroid and organoid models of BCa [24,25]. 

STAT3 is a transcription factor that plays a prominent role in tumorigenesis, making it a valuable target for cancer interception. According to various studies, STAT3 expression levels correlate with invasiveness and poor prognosis in BCa [26,27]. The development of effective inhibitors that target STAT3 signaling has received substantial attention. This study aimed to examine the anti-BCa effects of three promising small-molecule STAT3 inhibitors, i.e., GLG 302, TTI-101, and SH5-07, using various preclinical models. Despite the common target, these agents differ slightly in their molecular mechanism of action. GLG-302 inhibits STAT3 DNA-binding activity in vitro and decreases the proliferation of cells with constitutively active STAT3 [15]. TTI-101 is a competitive inhibitor of STAT3 and inhibits STAT3 recruitment and activation by targeting the receptor’s pY705 peptide-binding site in the SH2 domain [16]. Recently, SMARCB1 (switch/sucrose nonfermentable-related matrix-associated actin-dependent regulator of chromatin subfamily B member 1), which encodes INI-1 (integrase interactor 1) loss, was found to drive bladder tumor progression via activation of the IL6/STAT3 axis. In this context, pSTAT3 inhibition using TTI-101 was found to suppress the formation of in vivo tumor growth in SMARCB1 KO orthotopic cell line-derived xenografts and a xenograft-derived model from a SMARCB1-deficient patient [28].

SH5-07 is a hydroxamic acid analog that selectively inhibits STAT3 DNA-binding activity [17]. To determine the plausibility of these drugs in BCa cells, we examined the expression of STAT3 in BCa cells and found that all the cell line models expressed STAT3 and p-STAT3, thus allowing us to assess the anti-tumor effects of STAT3 inhibitors. Similar to earlier reports, we observed a strong anticancer effect of STAT3 inhibition [29]. Interestingly, the level of inhibition of the three agents correlated with target expression levels, with stronger inhibition of the J82 and NBT-II cell lines with higher levels of STAT3 compared to MB49. Comparing the size of spheroids and organoids exposed to a test compound is regarded as one of the best ways to assess the impact of a drug on tumor cells in a 3D environment [30,31]. Here, we observed a decrease in BCa spheroid and organoid size in response to STAT3 inhibitor treatment, implying that disrupting STAT3 inhibits cell proliferation in 3D cultures. This finding also supported the concept that reduced spheroid and organoid size correlated with decreased spheroid and organoid proliferation [32]. ATP is an important biochemical element in the tumor microenvironment, and its concentration influences tumor growth [33]. It is reported that the increased production of ATP accelerates the proliferation of cancer cells, which is strongly controlled by STAT3 activation [34]. 

In cells, mitochondria are believed to be the major source of ROS production during oxidative stress, and mitochondrial dysfunction is considered the earliest signal of cell death [35]. It is reported that STAT3 activation is inhibited by ROS production [36]. Interestingly, after treatment with TTI-101 and SH5-07, there was a significant increase in ROS production in BCa cells, indicating that it may also be involved in anticancer effects. Activated STAT3 signaling is known to regulate essential cancer cell mechanisms such as proliferation, survival, and metastasis pathways by modulating genes involved in cell proliferation (PCNA), the cell cycle (Cyclin D1), and apoptosis (PARP and Caspase 3) [37]. Numerous studies have shown that STAT3 activation requires phosphorylation of tyrosine 705 for homodimerization, nuclear translocation, and DNA-binding activity [38]. Here, we observed that TTI-101 and SH5-07 inhibit STAT3 phosphorylation, resulting in the suppression of STAT3-regulated gene products, including PCNA and Cyclin D1. The reduced expression of these two genes after treatment with TTI-101 and SH5-07 could provide an explanation for the anticancer effect of TTI-101 and SH5-07 by blocking STAT3 signaling. Apoptotic cell death is triggered by caspases, which cleave several proteins crucial for cellular survival and function [39]. PARP-1 is one of several known caspase cellular substrates. The cleavage of PARP-1 by caspases, especially caspase-3 and caspase-7, is thought to be a sign of apoptosis [40]. Our data showed that active caspase-3/7 and PARP cleavage remarkably increased after TTI-101 and SH5-07 treatment in BCa cell lines, spheroids, and organoids.

Several studies have reported that STAT3 signaling plays a key role in regulating CSCs, unique subpopulations of cells within tumors implicated in chemotherapy resistance [7]. Therefore, we examined the protein levels of the key stem cell markers CD44 and CD133 in BCa [41]. Upon TTI-101 and SH5-07 treatment, the expression of CD44 and CD133 was significantly reduced, whereas these markers were highly expressed in control spheroids and organoids. Overall, these results suggest that TTI-101 and SH5-07 may serve as novel therapeutic compounds that suppress CSC proliferation.

## 5. Conclusions

The present study showed that pharmacological targeting of p-STAT3 inhibited cell growth in multiple cell culture models of BCa. Our results demonstrated that TTI-101 and SH5-07 have anticancer efficacy against BCa, mediated by p-STAT3 inhibition, apoptosis induction, and stem cell depletion. Based on these encouraging in vitro findings, further in vivo investigations are needed to determine whether this agent could be clinically used for intercepting BCa recurrence and progression.

## Data Availability

The raw data supporting the conclusions of this article will be made available by the authors on request.
