# Correction: Singh et al. Stat3 Inhibitors TTI-101 and SH5-07 Suppress Bladder Cancer Cell Survival in 3D Tumor Models. *Cells* 2024, *13*, 1463

**DOI:** 10.3390/cells14040299

**Published:** 2025-02-18

**Authors:** Surya P. Singh, Gopal Pathuri, Adam S. Asch, Chinthalapally V. Rao, Venkateshwar Madka

**Affiliations:** 1 Center for Cancer Prevention and Drug Development, Stephenson Cancer Center, Hem-Onc Section, Department of Medicine, University of Oklahoma Health Sciences Center, Oklahoma City, OK 73104, USA; surya-singh@ouhsc.edu (S.P.S.); gopal-pathuri@ouhsc.edu (G.P.); cv-rao@ouhsc.edu (C.V.R.); 2 Stephenson Cancer Center, Hem-Onc Section, Department of Medicine, University of Oklahoma Health Sciences Center, Oklahoma City, OK 73104, USA; adam-asch@ouhsc.edu

In the original publication [1], there was a mistake in Figure 2 as published. In Figure 2A, the panels for 3 days and 12 days of observation were the same. The corrected Figure 2 appears below. The authors state that the scientific conclusions are unaffected. This correction was approved by the Academic Editor. The original publication has also been updated.

**Figure 2:**
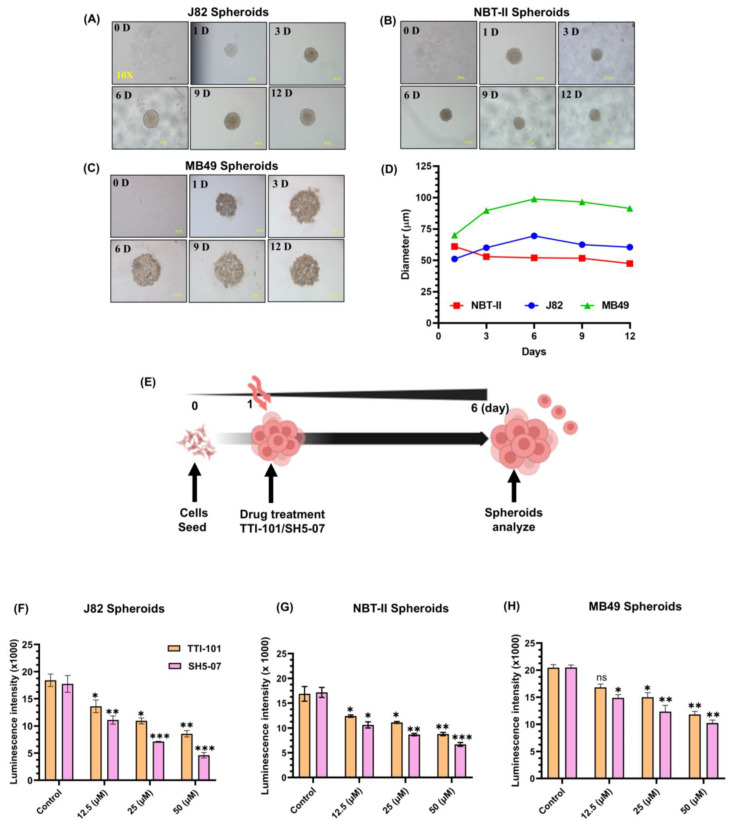

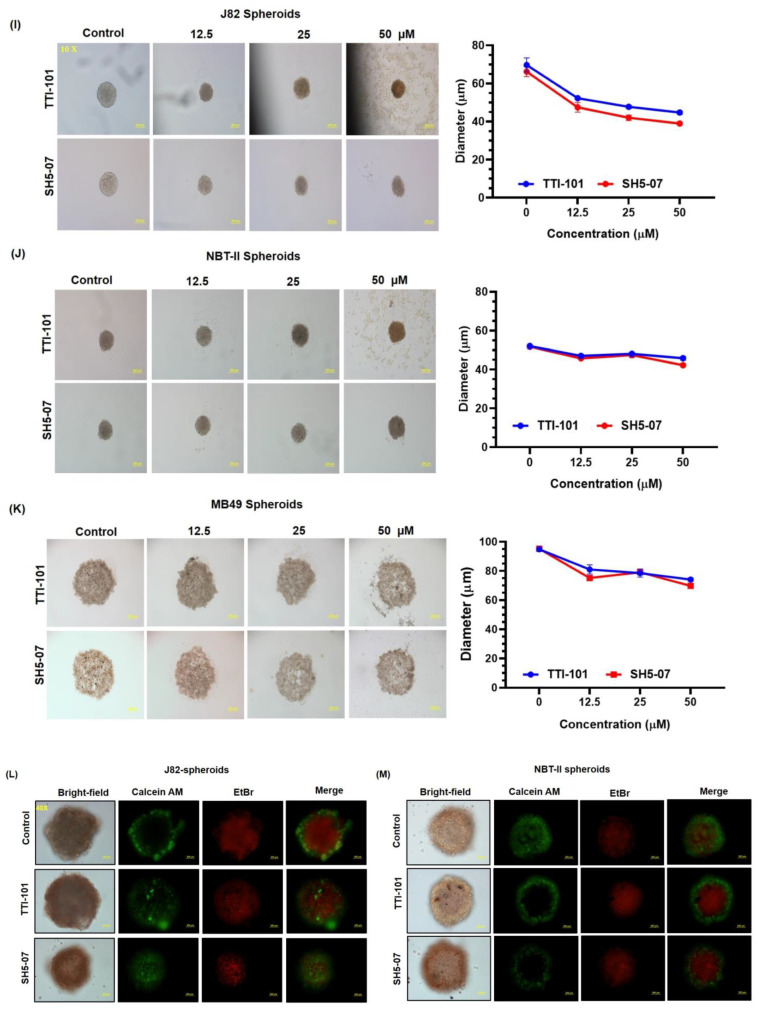
Effect of STAT3 inhibitors on proliferation of BCa spheroids. Representative images of spheroids generated using J82 (**A**), NBT-II (**B**), and MB49 (**C**) BCa cell lines. All images were captured at a 10X magnification. Growth curves of spheroid sizes (**D**). Schematic diagram illustrating treatment of spheroids with STAT3 inhibitors (**E**). Intracellular ATP content in (**I**–**K**) spheroids measured by luminescence on day six (**F**–**H**). Treatment with STAT3 inhibitors decreased the BCa spheroids size; bar represents 200 µM diameter (**I**–**K**). Spheroids were stained with calcein AM for live cells (green) and EtBr for dead cells (red). Representative images of control vs. treated BCa spheroids (**L**,**M**). All experiments were performed in triplicate (*n* = 3). Values are expressed as the mean ± SEM. Significance is indicated by * *p* < 0.05, ** *p* < 0.001, and *** *p* < 0.0001.
